# Macrophage-Related SPP1 as a Potential Biomarker for Early Lymph Node Metastasis in Lung Adenocarcinoma

**DOI:** 10.3389/fcell.2021.739358

**Published:** 2021-09-27

**Authors:** Bo Dong, Chunli Wu, Lan Huang, Yu Qi

**Affiliations:** ^1^Department of Thoracic Surgery, The First Affiliated Hospital of Zhengzhou University, Zhengzhou, China; ^2^Biotherapy Center, The First Affiliated Hospital of Zhengzhou University, Zhengzhou, China

**Keywords:** lung adenocarcinoma, early lymph node metastasis, SPP1, macrophage, M2 polarization, epithelial-mesenchymal transition (EMT)

## Abstract

Lymph node metastasis is a major factor that affects prognosis in patients with lung adenocarcinoma (LUAD). In some cases, lymph node metastasis has already occurred when the primary tumors are still small (i.e., early T stages), however, relevant studies on early lymph node metastasis are limited, and effective biomarkers remain lacking. This study aimed to explore new molecular biomarker for early lymph node metastasis in LUAD using transcriptome sequencing and experimental validation. Here, we performed transcriptome sequencing on tissues from 16 matched patients with Stage-T1 LUAD (eight cases of lymph node metastasis and eight cases of non-metastasis), and verified the transcriptome profiles in TCGA, GSE68465, and GSE43580 cohorts. With the bioinformatics analysis, we identified a higher abundance of M0 macrophages in the metastatic group using the CIBERSORT algorithm and immunohistochemistry (IHC) analysis and the enrichment of the epithelial–mesenchymal transition (EMT) pathway was identified in patients with higher M0 infiltration levels. Subsequently, the EMT hallmark gene *SPP1*, encoding secreted phosphoprotein 1 (SPP1), was identified to be significantly correlated with macrophage infiltration and M2 polarization, and was determined to be a key risk indicator for early lymph node metastasis. Notably, SPP1 in the blood, as detected by enzyme-linked immunosorbent assay (ELISA) showed a superior predictive capability for early lymph node metastasis [area under the curve (AUC) = 0.74]. Furthermore, a long non-coding RNA (lncRNA, AC037441), negatively correlated with SPP1 and macrophage infiltration, had also been identified and validated to be involved in the regulation of early lymph node metastasis. In conclusion, we revealed the potential role of macrophages in lymph node metastasis and identified the macrophage-related gene *SPP1* as a potential biomarker for early lymph node metastasis in LUAD.

## Introduction

Lung cancer is the most common form (11.6%) of malignant tumors worldwide (Bray et al., 2018; Ramos-Paradas et al., 2021). With advances in imaging technology and increased awareness of physical examinations, an increasing number of patients have been diagnosed with early-stage lung cancer. Lung adenocarcinoma (LUAD) represents a major form of lung cancer. Some LUAD patients experience lymph node metastasis or pathologically indistinguishable micrometastasis when the primary tumors are still small (that is, an early T-stage in the TNM staging system for lung cancer) (Martinez-Zayas et al., 2021), which seriously affects long-term survival (Allemani et al., 2018). The underlying mechanisms of early-stage metastasis require comprehensive exploration to identify effective biomarkers and optimize personalized cancer treatments.

The dynamic tumor immune microenvironment (TIME) plays a vital role in tumor proliferation, invasion, angiogenesis, and even immune escape (Altorki et al., 2019; Sautès-Fridman et al., 2019; Fane and Weeraratna, 2020). Studies exploring early lung cancer have shown that immunoediting is relatively common (Zhang et al., 2019b), and the immune system can induce a variety of routes resulting in immune evasion (Rosenthal et al., 2019). Single-cell analysis revealed that myeloid cell subsets might impair the anti-tumor immunity of T cells in early-stage LUAD (Lavin et al., 2017). In addition, the role of TIME in lymph node metastasis has been explored in breast cancer (Gu et al., 2019). Therefore, the relevant molecular mechanisms associated with early lymph node metastasis may be further elucidated in LUAD through the TIME analysis.

Macrophages differentiate from mononuclear phagocytic lineage cells, which reside in almost all tissues and participate in the immune response and homeostasis maintenance (Gentek et al., 2014). Macrophages can participate in tumorigenesis and progression (Qian and Pollard, 2010; Cassetta and Pollard, 2018; Wu et al., 2020). Deng et al. (2010) found that the abundant expression of tumor necrosis factor α (TNFα) and interleukin-6 (IL-6) by macrophages can create a favorable inflammatory environment for the occurrence of colonic adenocarcinomas. Moreover, macrophages play a considerable role in the acquisition of the epithelial– mesenchymal transition (EMT) hallmarks observed in gastric cancer tissues (Guan et al., 2021), pancreatic duct epithelial cells (Otto et al., 2021), and tracheal epithelial cells (Li et al., 2021), and EMT signatures plays a vital role in tumor metastasis (Lu and Kang, 2019). Macrophages are heterogeneous cell clusters with intricate functions and forms. The CIBERSORT algorithm can elucidate the gene expression characteristics of three macrophage subtypes: unpolarized M0 macrophages and polarized M1 (pro-inflammatory) and M2 (anti-inflammatory) macrophages (Newman et al., 2015). Higher M0 infiltration is closely related to unfavorable overall survival (OS) in LUAD patients (Yi et al., 2021; Zheng et al., 2021). However, the role of macrophages in early lymph node metastasis has not been elucidated.

Secreted phosphoprotein 1 (SPP1), a hallmark gene of the EMT process, can participate in extracellular matrix (ECM)-receptor interactions and the focal adhesion response pathway to regulate tumor metastasis and invasion. SPP1 is significantly associated with adverse survival outcomes in various cancers (Wei et al., 2020; He et al., 2021; Wang et al., 2021). Notably, higher SPP1 expression levels are related to a higher N stage in LUAD patients (Guo et al., 2020), although the underlying mechanism has not yet been studied fully. To explore the clinical phenomenon in which some LUAD patients develop lymph node metastasis at a very early T-stage, we revealed the crucial roles played by macrophages in lymph node metastasis and identified macrophage-related SPP1 as a potential biomarker for early metastasis in lung cancer.

## Materials and Methods

### Raw Data

To eliminate the influences of confounding factors (age, sex, and differentiation grade) on early lymph node metastasis, we selected eight patients with lymph node metastasis and matched eight patients without metastasis from the biobank of Stage-T1 LUAD. Transcriptome sequencing was performed on the tumor tissues and adjacent normal lung tissues from these 16 patients (one case of normal tissue was missing). RNA sequencing (RNA-seq) data and corresponding clinical information can be found in the supplementary materials. Furthermore, the transcriptome profiles of Stage-T1 LUAD patients from external validation cohorts were obtained from The Cancer Genome Atlas (TCGA) database (34 cases with lymph node metastasis, 131 cases without metastasis), the GSE68465 dataset (35 cases with lymph node metastasis, 114 cases without metastasis), and the GSE43580 dataset (11 cases of lymph node metastasis, 9 cases of no metastasis). The study protocol was approved by the institutional Ethics Committee of First Affiliated Hospital of Zhengzhou University (No. 2019-KY-255). Written informed consent was obtained from all study participants.

### Bioinformatics Analysis

The relative proportion of 22 immune cells were determined for Stage-T1 LUAD and adjacent lung samples using the CIBERSORT algorithm^1^. The algorithm of random sampling consisted of 1,000 permutations. Only samples with a *p*-value < 0.05 were included for subsequent analysis.

The weighted gene co-expression network analysis (WGCNA) package (Langfelder and Horvath, 2008) was used to construct scale-free networks and identify gene sets related to lymph node metastasis and macrophage infiltration (the minimum number of genes was set to 15, the mRNA scale-free network was formed when the soft threshold β was set to 10, and the long non-coding RNA (lncRNA) network was constructed when β = 8). Subsequently, hub RNAs in the modules were identified using Cystoscope software version 3.7.1.

### Enrichment Analysis of Biological Processes and Underlying Pathways

Gene Set Enrichment Analysis (GSEA) was conducted for on the hallmark gene sets of Molecular Signature Database (MSigDB) to explore the potential regulatory mechanisms associated with lymph node metastasis and M0 infiltration. GSEA analysis was performed using the software GESA version 4.01 (Subramanian et al., 2005), and gene sets related to EMT and inflammatory response (IR) hallmarks were also obtained from MSigDB.

Gene ontology (GO) and Kyoto Encyclopedia of Genes and Genomes (KEGG) enrichment analyses were performed using the clusterProfiler and enrichplot packages. Only terms for which both the *p*-value and q-value < 0.05 were considered significantly enriched.

### Enzyme-Linked Immunosorbent Assay

The plasma SPP1 levels in Stage-T1 LUAD patients were assayed using a commercial enzyme-linked immunosorbent assay (ELISA) kit (ELH-OPN-1; R&D company, United States). The 25-fold diluted plasma samples were directly transferred to a 96-well plate coated with an antibody specific for human SPP1 and assayed according to the manufacturer’s instructions. The absorbance value was recorded at 450 nm in a microtest plate spectrophotometer with the correction wavelength set at 540 nm. SPP1 levels were quantified using a calibration curve based on a human osteopontin standard. Both standards and samples were evaluated in duplicate, and the results were adopted only when the inter-assay variations were within the range provided by the manufacturer.

### Quantitative Reverse Transcriptase-Polymerase Chain Reaction

Total RNA was extracted from 25 other tumor samples (12 cases of lymph node metastasis, 13 cases of non-metastasis) in the biobank of Stage-T1 LUAD using NcmZol Reagent (M5100; NCM Biotech, China), according to the manufacturer’s protocol, and cDNA was synthesized using the Reverse Transcription Kit (R2020; US Everbright^®^ Inc., China). For quantitative reverse transcriptase-polymerase chain reaction (qRT-PCR) analysis, PCR was performed using a reaction mixture containing a cDNA template, primers, and a Universal SYBR Green qPCR SuperMix (S2024; US Everbright^®^ Inc.) in a Step One Plus Real-Time PCR System (Thermo Fisher Scientific). The primers for SPP1 were 5′-ACAGCCAGGACTCCATTGAC-3′ and 5′-GGGGACAACTGGAGTGAAAA-3′, and the primers for the lncRNA (AC037441) were 5′-TCACTGAGCAGGGTTCACAC-3′, and 5′-TCTTCACTGGCCTCCAAAAT-3′.

### Immunohistochemical

Immunohistochemical (IHC) staining was performed in paraffin-embedded continuous tissue sections. Tissue sections were dewaxed and rehydrated with xylene and gradient alcohol washes, and antigen retrieval was then performed at 121°C for 1 min using pH 6.0 citrate buffer. Subsequently, sections were blocked with hydrogen peroxide for 20 min at room temperature and incubated with primary antibodies against CD68 (1:200; GB14043, Service bio, China), CD163 (1:500; GB113152, Service bio), inducible nitric oxide synthase (iNOS; 1:1,000; GB11119, Service bio), interleukin (IL)-1β (1:800; GB11113, Service bio), and SPP1 (1:100; A1499; Abcam, United Kingdom) overnight at 4°C. After incubation with the secondary antibody at room temperature for 1 h, immunostaining with DAB and counterstaining of the slide with hematoxylin, the entire stained sections were scanned and analyzed in a panoramic view. After scanning the entire sections with a tissue scanner, the Seville image analysis system was used to identify and calculate the total tissue area, the stained tissue area, and the staining intensity score (0 = no color; 1 = faint yellow; 2 = light brown; and 3 = dark brown) and the percentage of various staining intensity cells under the panoramic view. CD68 antibody was used as a marker for macrophages; iNOS-positive cells and IL-1β-positive cells were regarded as M1 macrophages; and CD163−positive cells were regarded as M2 macrophages. In addition, the immunohistochemical staining of CD68/CD163/iNOS/IL-1β was quantified by measuring the positive area ratio (stained tissue area/total tissue area). SPP1 was quantified with a histochemistry score (H-score) to examine differences between samples from the early lymph node metastasis and non-metastasis groups [H-Score = Σ (percentage of staining intensity cells × staining intensity)].

### Statistical Analysis

Statistical analyses were performed using R 4.0.3, GraphPad Prism 9.0, and SPSS 22.0. The expression levels of SPP1 and macrophage-related markers were compared between groups by an unpaired *t*-test. Survival analysis was performed using “survival” and “survminer” R packages. The Kaplan–Meier method was used to generate survival curves, and the log-rank test was used to determine significant differences in survival. The median values of SPP1 expression, M0 and M2 infiltration levels were set as the cut-off point for grouping. The correlation between SPP1 expression level and the abundance of immune cell infiltration was analyzed by Pearson’s correlation test. The predictive performance of SPP1 detected in tissues and plasma for predicting early lymph node metastasis was evaluated using receiver operator characteristic (ROC) curves. *p* < 0.05 was considered significant.

## Results

### M0 Macrophages Present a High Infiltration Level in Lung Adenocarcinoma Patients With Early Lymph Node Metastasis

**FIGURE 1:**
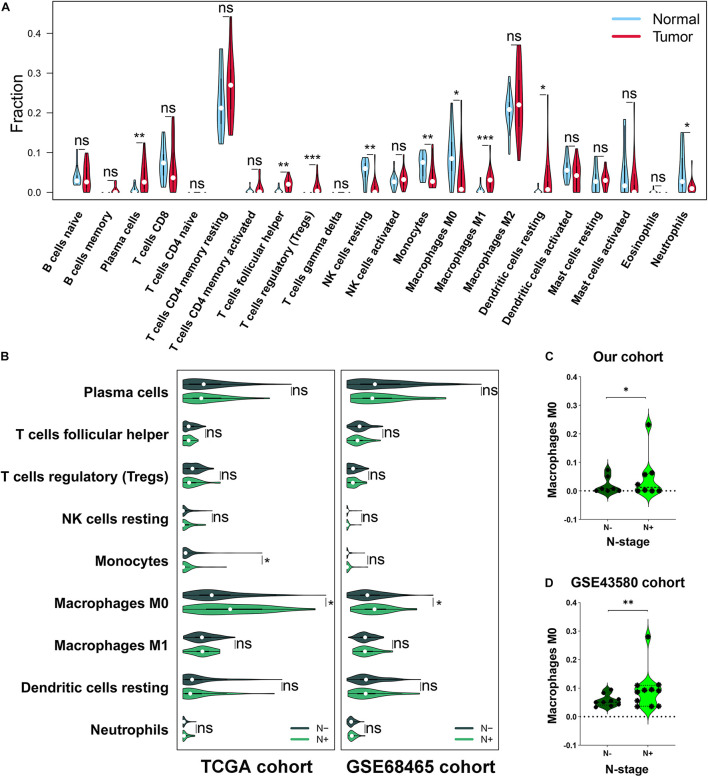
Analysis of the immune cell infiltration abundance in Stage-T1 lung adenocarcinoma (LUAD). **(A)** Comparison of the infiltration levels of 22 immune cells in tumor tissues versus adjacent lung tissues. **(B)** Comparison of the infiltration abundance of nine immune cell types in the lymph node metastasis group versus the non-metastasis group of TCGA and GSE68465 cohorts. In our cohort **(C)** and the GSE43580 cohort **(D)**, a higher M0 infiltration was associated with early lymph node metastasis. ns, not significant, *p < 0.05, **p < 0.01, and ***p < 0.001. TCGA, The Cancer Genome Atlas.

First, based on the analysis of infiltration abundance among immune cells in the 16 cases of Stage-T1 LUAD tissues and the 15 cases of paired normal adjacent tissues, nine immune cell subtypes were found to have distinct infiltration levels (Figure 1A). The infiltration of plasma cells (*p* < 0.01), follicular helper T cells (FTH; *p* < 0.01), regulatory T cells (Tregs; *p* < 0.001), M1 macrophages (*p* < 0.001), and resting dendritic cells (*p* < 0.05) in tumor tissues was higher than that in normal lung tissues, whereas resting natural killer cells (*p* < 0.01), monocytes (*p* < 0.01), M0 macrophages (*p* < 0.05), and neutrophils (*p* < 0.05) had lower infiltration levels. These results suggested that the TIME presented a wide range of variability in early LUAD.

To identify the TIME characteristics associated with early lymph node metastasis in LUAD, the infiltration abundances of the nine immune cell subtypes were further analyzed in a TCGA cohort (*n* = 165) and a GSE68465 cohort (*n* = 149). M0 macrophages identified as highly infiltrated in the metastatic group (Figure 1B, *p* < 0.05), which was further validated in our cohort (*n* = 16, *p* < 0.05, Figure 1C) and the GSE43580 cohort (*n* = 20, *p* < 0.01, Figure 1D). Through the joint analysis of four independent cohorts, we demonstrated a correlation between M0 infiltration and early lymph node metastasis in LUAD.

### Gene Set Enrichment Analysis and the Weighted Gene Co-expression Network Analysis Algorithm Identified Gene Sets Related to Lymph Node Metastasis and M0 Infiltration

**FIGURE 2:**
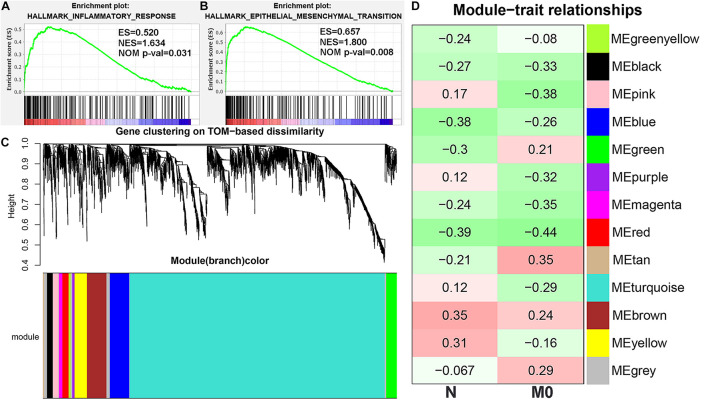
Identification of mRNA modules associated with lymph node metastasis and M0 infiltration. Gene set enrichment analysis (GSEA) suggested that early lymph node metastasis was related to the inflammatory response (IR) pathway **(A)**, and the epithelial–mesenchymal transition (EMT) pathway were significantly enriched in the M0 high-infiltration group **(B)**. **(C)** The scale-free mRNA network was clustered into 13 modules by average hierarchical clustering and dynamic tree clipping. **(D)** The correlation heatmap was used to describe the relationship between 13 genesets and N stage, M0 infiltration. ES, enrichment score. NES, normalized ES. NOM *p*-val, normalized *p*-value. TOM, topological overlap matrix.

To further explore the underlying mechanisms of M0 infiltration and early lymph node metastasis, GSEA was performed in our cohort, indicating that IR hallmarks were associated with early lymph node metastasis (Figure 2A), and the EMT pathway was significantly enriched in the M0 high-infiltration group (Figure 2B). Moreover, the WGCNA algorithm was applied to construct an mRNA scale-free network, which resulted in the definition of 13 modules by average hierarchical clustering and dynamic tree clipping (Figure 2C). The black, blue, magenta, and red modules were negatively correlated with N stages and M0 infiltration, whereas the brown module showed a consistent positive correlation (Figure 2D).

**FIGURE 3:**
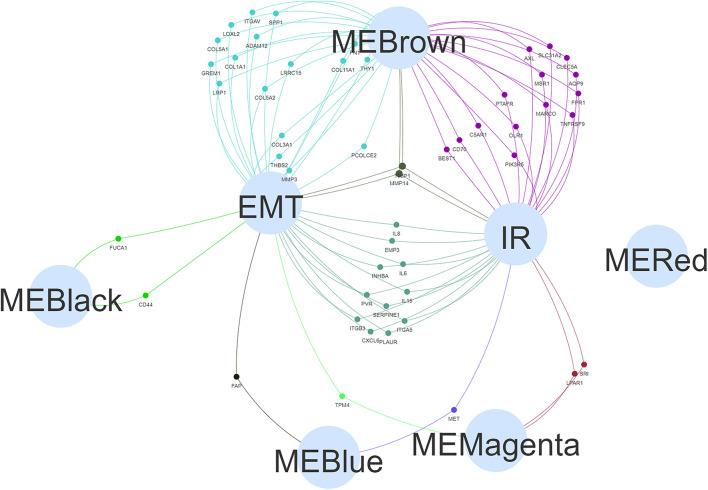
The Venn diagram showing the intersection of five module genesets (“MEBrown,” “MEBlack,” “MERed,” “MEBlue,” and “MEMagenta”) and hallmark genesets [epithelial–mesenchymal transition (EMT) and inflammatory response (IR)].

Finally, a Venn diagram was constructed to investigate the intersections among the genes in the five modules and the genes identified as EMT and IR hallmarks (the hallmark gene sets were obtained from MSigDB). Compared with other modules, the brown module had more intersections with EMT and IR hallmarks and was selected for further analysis (Figure 3).

### The Enrichment Analysis of the Selected Gene Suggests Cell Adhesion Plays a Key Role in Early Lymph Node Metastasis

**FIGURE 4:**
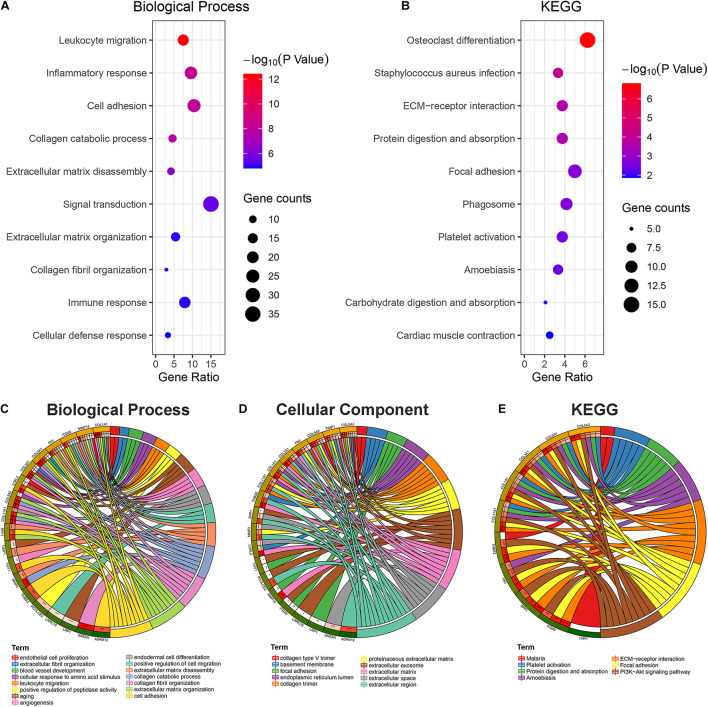
Gene ontology (GO) and Kyoto Encyclopedia of Genes and Genomes (KEGG) enrichment analysis. The bubble diagram displayed the biological function **(A)** and KEGG **(B)** enrichment analysis results of the brown module genes. Biological function **(C)**, cellular component **(D)**, and KEGG **(E)** enrichment analysis was performed on 19 epithelial–mesenchymal transition (EMT)-related genes in the brown module, and the chord diagram was used to show the enriched GO/KEGG terms and statistical differences. *p < 0.05, **p < 0.01, and ***p < 0.001.

The biological processes associated with genes in the brown module were primarily mapped to signal transduction, cell adhesion, inflammation, and immune response (Figure 4A). The KEGG enrichment analysis indicated the significant enrichment of focal adhesion and ECM-receptor interactions (Figure 4B). In addition, to further elucidate the potential regulatory mechanisms associated with the EMT during early lymph node metastasis, 19 EMT-related genes in the brown module were specifically selected for enrichment analysis. The results suggested that these 19 genes were primarily associated with cell adhesion and extracellular matrix organization (Figure 4C) and were enriched in the extracellular region (Figure 4D) and the phosphoinositide 3-kinase (PI3K)-AKT signaling pathway (Figure 4E). These clues indicated that the changes in cell adhesion might be key factors in early lymph node metastasis.

### The Epithelial–Mesenchymal Transition Hallmark Gene Secreted Phosphoprotein 1 Is Associated With Early Lymph Node Metastasis and M0 Infiltration as Validated by Three External Cohorts

**FIGURE 5:**
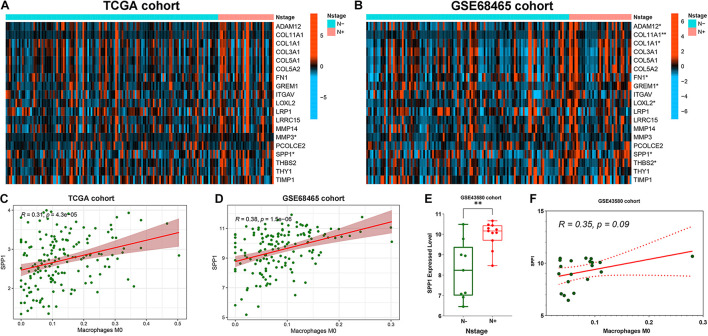
Correlation analysis of secreted phosphoprotein 1 (SPP1) with early lymph node metastasis and macrophage infiltration in Stage-T1 lung adenocarcinoma (LUAD) patients from three external cohorts (TCGA, GSE68465 and GSE43580 cohorts). **(A)** SPP1 and matrix metalloprotease 3 (MMP3) were associated with N stages in the TCGA cohort. **(B)** thrombospondin 2 (THBS2), SPP1, lysyl oxidase like 2 (LOXL2), gremlin 1 (GREM1), fibronectin 1 (FN1), collagen type I alpha 1 chain (COL1A1), COL11A1, and ADAM metallopeptidase domain 12 (ADAM12) were associated with N stages in the GSE68465 cohort. TCGA cohort **(C)** and GSE68465 cohort **(D)** analysis verified that SPP1 was significantly positively correlated with M0 infiltration. In the GSE43580 cohort, SPP1 was highly expressed in the lymph node metastasis group **(E)**, and the correlation between SPP1 and M0 was also investigated **(F)**. *p < 0.05, **p < 0.01.

The association of 19 EMT-related genes with lymph node metastasis and M0 infiltration was further analyzed in three external cohorts. First, SPP1 was identified through the expression level analysis of 19 genes comparing the lymph node metastasis group with the non-metastatic group in both TCGA (*p* < 0.05, Figure 5A) and GSE68465 cohorts (*p* < 0.05, Figure 5B). Subsequently, the correlation between SPP1 expression and M0 infiltration was further verified in TCGA cases (*r* = 0.31, *p* < 0.001, Figure 5C) and the GSE68465 cohort (*r* = 0.38, *p* < 0.001, Figure 5D). In the GSE43580 cohort, SPP1 was highly expressed in the lymph node metastasis group (*p* < 0.01, Figure 5E), and the correlation between SPP1 and M0 was also analyzed (*r* = 0.35, *p* = 0.09, Figure 5F). Our results suggested that macrophage-related SPP1 expression may be a crucial risk indicator for early lymph node metastasis in LUAD.

### Secreted Phosphoprotein 1 Could Predicts Early Lymph Node Metastasis and Affects Prognosis in Stage-T1 Lung Adenocarcinoma Patients, Which May Be Related to the M2 Polarization

**FIGURE 6:**
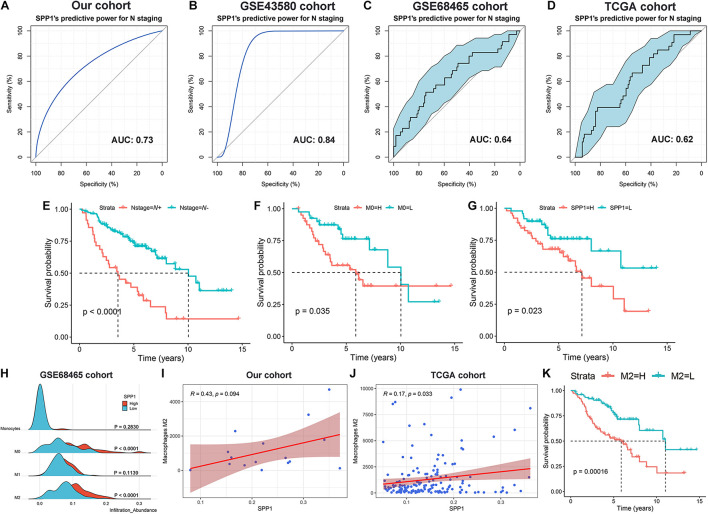
Analysis of SPP1 for the prediction of lymph node metastasis and influence on the long-term survival of Stage-T1 LUAD patients. The receiver operating curve (ROC) and area under the curve (AUC) were used to evaluate the predictive power of SPP1 for early lymph node metastasis in our cohort [**(A)** *n* = 16], GSE43580 cohort [**(B)** *n* = 20], GSE68465 cohort [**(C)** *n* = 149] and TCGA cohort [**(D)** *n* = 165]. Kaplan–Meier curves of overall survival for patients with distinct N stages **(E),** M0 infiltration **(F),** and SPP1 expression **(G)**. **(H)** Multipeaked mountain map displaying the infiltration abundance divergence among the monophagocytic system (Monocytes, M0, M1, and M2) across groups with different levels of SPP1 expression in GSE68465 cohort. The correlation analysis of M2 infiltration and SPP1 expression in our cohort **(I)** and TCGA cohort **(J)**. **(K)** Kaplan–Meier curve to evaluate the influence of M2 infiltration on the OS in patients with Stage-T1 LUAD.

The predictive efficiency of SPP1 for early lymph node metastasis was evaluated in four cohorts. In our patient cohort (*n* = 16, Figure 6A) and the GSE43580 cohort (*n* = 20, Figure 6B), the area under the ROC curve (AUC) values were 0.73 and 0.84, respectively. The AUCs for the GSE68465 (*n* = 149, Figure 6C) and TCGA cohorts (*n* = 165, Figure 6D) were 0.64 (95% CI: 0.53–0.75) and 0.62 (95% CI: 0.52–0.72), respectively. In addition, using the survival data for the GSE68465 cohort, we demonstrated that early lymph node metastasis (*p* < 0.001, Figure 6E), higher M0 infiltration (*p* = 0.035, Figure 6F), and higher SPP1 expression (*p* = 0.023, Figure 6G) were significantly associated with the poor prognosis among patients with Stage-T1 LUAD.

The correlation between SPP1 and the monophagocytic system was systematically explored in early LUAD. In the GSE68465 cohort, the M0 and M2 infiltration abundances were significantly increased in the SPP1 high expression group (*p* < 0.001), whereas differences in monocyte (*p* = 0.283) and M1 infiltration (*p* = 0.114) were not significant between groups (Figure 6H). The correlation between SPP1 expression and M2 infiltration was verified using data from the cohorts from TCGA (*r* = 0.17, *p* = 0.033, Figure 6I) and our patients (*r* = 0.43, *p* = 0.094, Figure 6J). Patients with higher M2 infiltration had a shorter OS (*p* < 0.001, Figure 6K). Therefore, we speculated that SPP1 might induce the M2 polarization of macrophages in Stage-T1 LUAD, which could be closely related to the long-term survival of patients.

### The lncRNA AC037441 Is Associated With Early Lymph Node Metastasis, M0 Infiltration, and Secreted Phosphoprotein 1 Expression

**FIGURE 7:**
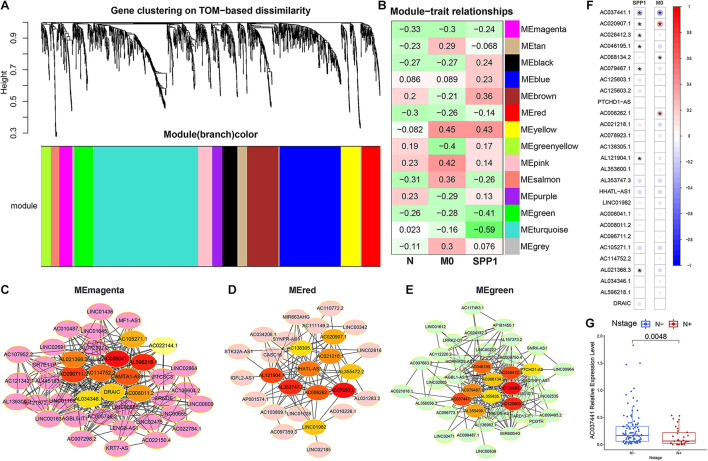
Identification of long non-coding RNAs (lncRNAs) associated with early lymph node metastasis, SPP1 expression, and M0 infiltration. **(A)** Hierarchical clustering analysis was performed to determine the lncRNA co-expression modules represented by different colors. **(B)** The heatmap was used to demonstrate the correlation between the modules and lymph node metastasis, SPP1 expression, and M0 infiltration. **(C–E)** The top 10 hub lncRNAs and their first-level nodes of 3 key modules (“MEmagenta,” “MEred,” and “MEgreen”) were identified through the maximal clique centrality (MCC) algorithm (the color of the hub nodes represented the hub level, red is the highest, and followed by orange). **(F)** The correlation analysis of 30 hub lncRNAs with SPP1 expression and M0 infiltration is based on TCGA dataset. **(G)** Expression level analysis of lncRNA (AC037441) between the lymph node metastasis group and the non-metastatic group in TCGA cohort. *p < 0.05.

To explore the potential lncRNA/SPP1-macrophage axis associated with early lymph node metastasis, the WGCNA algorithm was used to construct an lncRNA co-expression network based on our cohort. Thirteen modules were identified by subsequent hierarchical clustering and dynamic tree clipping (Figure 7A), among which the magenta, red and green modules showed consistent negative correlations with early lymph node metastasis, M0 infiltration, and SPP1 expression (Figure 7B). The maximal clique centrality (MCC) algorithm was used to determine hub lncRNAs for three modules (Figures 7C–E). In addition, further verification using TCGA cohort data identified that the hub lncRNA (AC037441) was significantly negatively correlated with SPP1 (*r* = −0.27, *p* < 0.05) and M0 infiltration (*R* = –0.31, *p* < 0.05; Figure 7F), and the expression level of AC037441 in the early lymph node metastasis group was significantly lower than that in the non-metastatic group (*p* = 0.005, Figure 7G). The analysis of the two cohorts revealed a potential RNA-immune cell axis (lncRNA AC037441/SPP1-macrophage) associated with early lymph node metastasis. However, further cell biological validation remains necessary.

### Macrophage-Related Secreted Phosphoprotein 1Is a Biomarker for Early Lymph Node Metastasis, Validated in Tissue and Plasma Samples From Stage-T1 Lung Adenocarcinoma Patients

**FIGURE 8:**
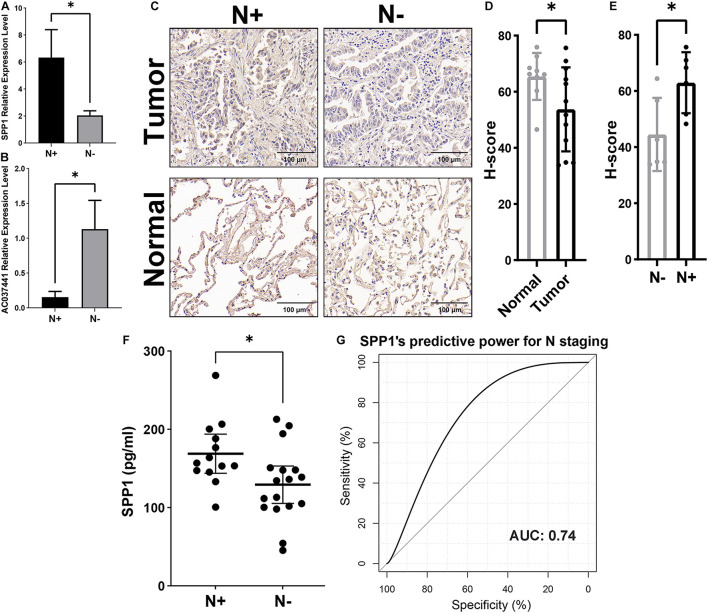
Validation of SPP1 as a potential biomarker for early lymph node metastasis by quantitative reverse transcriptase-polymerase chain reaction (qRT-PCR), immunohistochemical (IHC), and enzyme-linked immunosorbent assay (ELISA) assays. The expression levels of SPP1 **(A)** and lncRNA (AC037441) **(B)** in tumor samples were compared by qRT-PCR in the lymph node metastasis group versus the non-metastatic group. **(C)** The representative IHC figures of SPP1 in Stage-T1 LUAD patients with and without lymph node metastasis. **(D)** The H-scores of SPP1 in tumor tissues (*n* = 12) were lower than that of adjacent normal tissues (*n* = 9). **(E)** The tumor sample scores distinction of SPP1 in the lymph node metastatic versus non-metastatic groups. **(F)** Comparison of the SPP1 concentration in the plasma of Stage-T1 LUAD patients between patients with lymph node metastasis and those without metastasis. **(G)** The receiver operating curve (ROC) and area under the curve (AUC) were used to evaluate the predictive efficiency of plasma SPP1 concentrations for early lymph node metastasis. *p < 0.05.

Following the bioinformatics analysis, the findings were verified using tissue and plasma samples from Stage-T1 LUAD patients. The qRT-PCR assay was performed on 25 cases of tumor tissues (12 cases of lymph node metastasis, 13 cases of non-metastasis), we confirmed the significantly increased expression of SPP1 (Figure 8A, *p* < 0.05) and the significantly decreased expression of lncRNA (AC037441) (Figure 8B, *p* < 0.05) in the lymph node metastasis group. The IHC analysis of 12 tumor samples (six cases of lymph node metastasis and six cases of non-metastasis) and nine paracancerous samples showed that SPP1 had lower H-scores in tumor tissues (Figures 8C,D, *p* < 0.05), and the H-score of the lymph node metastatic group was significantly higher than that of the non-metastatic group (Figures 8C,E, *p* < 0.05). The SPP1 concentration in plasma was quantified by ELISA, which showed SPP1 concentrations in patients with lymph node metastasis were significantly higher than in the non-metastatic group (Figure 8F, *p* < 0.05), Moreover, the ROC curve showed that the plasma SPP1 level had good efficiency for predicting early lymph node metastasis (AUC = 0.74, Figure 8G).

**FIGURE 9:**
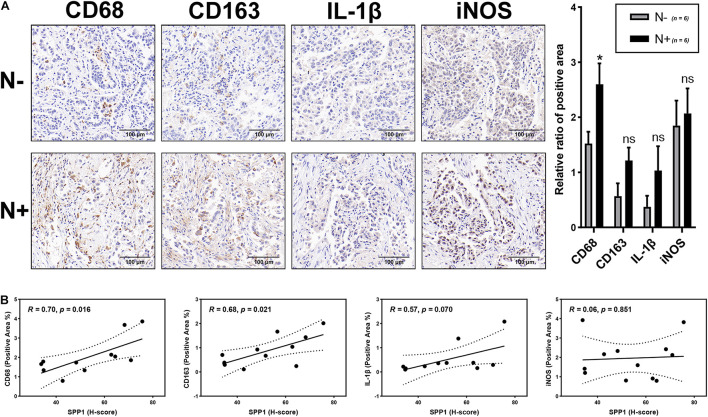
Correlation analysis of macrophages with lymph node metastasis and SPP1 expression. **(A)** The representative figures of macrophage markers (CD68, CD163, IL-1β, and iNOS) staining in Stage-T1 LUAD samples and the comparison of positive area ratio in the lymph node metastatic and non-metastatic groups. **(B)** The H-score of SPP1 was significantly positively correlated with the positive area ratio of CD68 (*R* = 0.70, *p* = 0.016) and CD163 (*R* = 0.68, *p* = 0.021), while the correlation with the positive area ratio of IL-1β (*R* = 0.57, *p* = 0.070) and iNOS (*R* = 0.06, *p* = 0.851) was not statistically significant.*p < 0.05.

Finally, the correlations between macrophages and lymph node metastasis and SPP1 expression were validated through the IHC analysis of tumor samples. The results showed that the positive area of macrophage marker (CD68) staining significantly increased in the lymph node metastasis group compared with the non-metastatic groups (*p* < 0.05), whereas the staining for M2 (CD163) and M1 markers (IL-1β and iNOS) showed no significant differences (*P* > 0.05; Figure 9A). In addition, the H-score of SPP1 was significantly positively correlated with the positive areas of CD68 staining (*r* = 0.70, *p* = 0.016) and CD163 staining (*r* = 0.68, *p* = 0.021); however, no significant correlation was observed for the positive areas of IL-1β staining (*r* = 0.57, *p* = 0.070) and iNOS staining (*r* = 0.06, *p* = 0.851; Figure 9B).

## Discussion

The TIME can regulate the growth and metastasis of lung cancer by promoting inflammation, angiogenesis, and immune modulation (Altorki et al., 2019; Bonanno et al., 2019; Rosenthal et al., 2019). In this study, we found that the infiltration of plasma cells, FTH, Tregs, M1 macrophages, and resting dendritic cells in LUAD tissues were higher than those in normal lung tissues, whereas resting natural killer cells, monocytes, M0 macrophages, and neutrophils had lower infiltration levels. Our results further demonstrated the diverse TIME in LUAD. In addition, the correlation between the TIME and lymph node metastasis has been reported. Coffelt et al. (2015) demonstrated that γδ T cell and neutrophil infiltration play important roles in the lymph node metastasis of breast cancer. However, the TIME changes that occur during LUAD lymph node metastasis have not been studied. We revealed a significant correlation between macrophages and lymph node metastasis through bioinformatics analysis and experimental verification. The role of macrophages in tumor metastasis has aroused broad concern, studies have indicated that macrophages can promote the metastasis of colorectal cancer cells by secreting miRNA-containing exosomes (Li et al., 2019) or cytokines, including IL-10 and IL-17 (Lan et al., 2019).

Notably, some LUAD patients develop lymph node metastasis when the diameter of the primary tumor remains very small. Early lymph node metastasis significantly affects the long-term survival of patients (Liu et al., 2019; Zhang et al., 2019a), and our study verified that stage-T1 patients with lymph node metastasis have a shorter OS. (Takada et al., 2020) demonstrated that early lymph node metastasis in breast cancer is related to tumor-infiltrating lymphocytes, whereas no relevant research has been published on early lymph node metastasis of lung cancer. In our study, GSEA showed that the IR pathway was significantly enriched in the metastasis group. IR plays an important role in tumorigenesis, progression, and metastasis (Diakos et al., 2014; Crusz and Balkwill, 2015). Moreover, the IR can also be modulated by pro-inflammatory cytokines and chemokines secreted by activated immune cells (Deng et al., 2010; Ferrari et al., 2019).

The EMT process can endow tumor cells with aggressiveness and mobility, which can be regarded as a sign of cancer metastasis (Lu and Kang, 2019) and poor prognosis (Fazilaty et al., 2019). We found that the EMT pathway was significantly enriched in the Stage-T1 LUAD patients with higher macrophage infiltration abundance. Wei C. et al. (2019) revealed that macrophages could secrete IL-6 to regulate the EMT program of colorectal cancer cells. Studies have also shown that EMT-related gene SPP1 is a potent macrophage chemokine, and SPP1 blockade can impair the macrophage recruitment ability of tumors (Wei J. et al., 2019). In this study, SPP1 was identified based on the exploration of the EMT hallmarks and macrophage-related genes in multiple cohorts, and was validated as a potential biomarker for early lymph node metastasis based on the tissue and plasma samples. In addition, SPP1 was shown to be significantly related to M2 macrophage infiltration in early LUAD, which was significantly associated with poor prognosis. Zhu et al. (2019) demonstrated that the number of tumor-associated macrophages and the expression level of M2 markers decreased significantly in tumor tissues from SPP1 knockout mice compared with those from the control group, and the effect of SPP1 on the M2 phenotype maintenance (Wei J. et al., 2019) and M2 polarization (Zhang et al., 2017) had also been proved in many studies. Furthermore, the inhibition of SPP1 protein activation can prevent lung cancer metastasis by inactivating integrin/CD44-associated signaling and rearranging the actin cytoskeleton (Chiou et al., 2019). However, the correlation between SPP1 and tumor lymph node metastasis has not yet been investigated.

It has been well documented that M2 macrophages promote tumor growth and metastasis (Tao et al., 2020). On the other hand, many studies indicated tumor cells stimulate naive M0 macrophages to differentiate into M2 macrophages (Liu et al., 2021). Macrophages are mainly M2 subtypes in tumor microenvironment, especially in advanced stage of tumors. However, the characteristics of macrophage subsets remains unclear during the early stage of tumor metastasis. Interestingly, we found high M0 infiltration in LUAD with early lymph node metastasis. Our results suggest that more recruitment of naive M0 macrophages and subsequent M2 polarization in LUAD are likely a key determinant for early lymph node metastasis. Finally, a potential lncRNA (AC037441)/SPP1-macrophage axis was identified through the application of the WGCNA algorithm and correlation analysis, which has not been previously reported. Moreover, a significant correlation between lncRNA (AC037441) and early lymph node metastasis was further confirmed experimentally.

Some limitations must be addressed in this study. First, although a multi-cohort analysis and related experimental validation were conducted, the number of patients with Stage-T1 LUAD was relatively small, and more samples remain necessary for further verification. In addition, the relationship between SPP1 and macrophage infiltration was primarily based on correlation studies, and additional biological experiments remain necessary to explore the detailed underlying mechanism.

In summary, a correlation between macrophages and early lymph node metastasis in Stage-T1 LUAD was identified through a multi-cohort analysis and experimental validation. Patients with high macrophage infiltration were showed to present the significant enrichment of EMT pathway, and SPP1, an EMT hallmark gene, was identified to be significantly associated with macrophage infiltration and lymph node metastasis. In addition, the predictive efficacy of SPP1 detection in plasma for early lymph node metastasis was evaluated. Our study explored a potential mechanism for early lymph node metastasis in LUAD and identified and verified SPP1 as a potential biomarker.

## Data Availability Statement

Transcript sequencing data and clinical information for the 31 patient samples analyzed in this study can be found in the Supplementary Material. In addition, publicly available datasets were analyzed in this study, which can be found at TCGA (https://www.cancer.gov/about-nci/organization/ccg/research/structural-genomics/tcga) and GEO omnibus (https://www.ncbi.nlm.nih.gov/geo/) (GSE68465 and GSE43580).

## Ethics Statement

The studies involving human participants were reviewed and approved by the institutional Ethics Committee of First Affiliated Hospital of Zhengzhou University. The patients/participants provided their written informed consent to participate in this study.

## Author Contributions

YQ and LH: conceptualization, writing—review and editing, and funding acquisition. BD: methodology. BD and CW: formal analysis and writing—original draft preparation. CW: data curation. YQ: supervision and project administration. All authors contributed to the article and approved the submitted version.

## Conflict of Interest

The authors declare that the research was conducted in the absence of any commercial or financial relationships that could be construed as a potential conflict of interest.

## Publisher’s Note

All claims expressed in this article are solely those of the authors and do not necessarily represent those of their affiliated organizations, or those of the publisher, the editors and the reviewers. Any product that may be evaluated in this article, or claim that may be made by its manufacturer, is not guaranteed or endorsed by the publisher.

## Footnotes

1
https://cibersort.stanford.edu/
